# Subclinical left ventricular dysfunction in rheumatoid arthritis: findings from the prospective Porto-RA cohort

**DOI:** 10.1007/s00392-024-02548-6

**Published:** 2024-09-30

**Authors:** André Alexandre, David Sá-Couto, Mariana Brandão, Sofia Cabral, Tomás Fonseca, Rita Quelhas Costa, António Marinho, Carlos Vasconcelos, Betânia Ferreira, João Pedro Ferreira, Patrícia Rodrigues

**Affiliations:** 1 Department of Cardiology, Unidade Local de Saúde de Santo António, Largo Do Prof. Abel Salazar, 4099-001 Porto, Portugal; 2 ICBAS—School of Medicine and Biomedical Sciences, University of Porto, 4050-313 Porto, Portugal; 3 Clinical Immunology Unit, Unidade Local de Saúde de Santo António, 4099-001 Porto, Portugal; 4 Autoimmunity and Neurosciences Group, UMIB—Unit for Multidisciplinary Research in Biomedicine, ICBAS—School of Medicine and Biomedical Sciences, University of Porto, 4050-313 Porto, Portugal; 5 Department of Internal Medicine, Unidade Local de Saúde de Entre Douro E Vouga, Aveiro, Portugal; 6 Hospital da Luz Arrábida, Vila Nova de Gaia, Portugal; 7 Université de Lorraine, INSERM, Centre d’Investigations Cliniques Plurithématique 1433, CHRU de Nancy, Inserm U1116 F-CRIN INI-CRCT (Cardiovascular and Renal Clinical Trialists), Nancy, France; 8 Department of Surgery and Physiology, Faculty of Medicine of the University of Porto, Porto, Portugal; 9 Cardiovascular Research Group, UMIB—Unit for Multidisciplinary Research in Biomedicine, ICBAS—School of Medicine and Biomedical Sciences, University of Porto, 4050-313 Porto, Portugal

**Keywords:** Rheumatoid arthritis, Major adverse cardiovascular events, Left ventricular global longitudinal strain, Subclinical cardiac dysfunction, Heart failure

## Abstract

**Aim:**

Patients with rheumatoid arthritis (RA) have an increased risk of cardiac dysfunction and heart failure (HF) due to a pro-inflammatory state. Detecting cardiac dysfunction in RA is challenging as these patients often present preserved ejection fraction (EF) but may have subclinical ventricular dysfunction. Echocardiographic strain analysis is a promising tool for early detection of subclinical left ventricular systolic dysfunction (LVSD). This study assesses the prognostic role of strain analysis in RA.

**Methods and results:**

Prospective study of 277 RA patients without known heart disease and preserved EF, categorized by left ventricular global longitudinal strain (GLS): normal GLS (≤ − 18%) vs. subclinical LVSD (> − 18%). Primary outcome was a composite of myocardial infarction, HF hospitalization, stroke, or cardiovascular death (MACE). Mean age was 57 years, 79% female. Although mean GLS was within normal (− 20 ± 3%), subclinical LVSD was observed in 24% of patients (*n* = 67) and was positively correlated with older age (OR 1.54 per 10 years; *p* < 0.001) and comorbid conditions, such as dyslipidemia (OR 2.27; *p* = 0.004), obesity (OR 2.29; *p* = 0.015), and chronic kidney disease (OR 8.39; *p* = 0.012). Subclinical LVSD was independently associated with a 3.9-fold higher risk of MACE (*p* = 0.003) and a 3.4-fold higher risk of HF hospitalization/cardiovascular death (*p* = 0.041). A GLS threshold of > − 18.5% provided optimal sensitivity (78%) and specificity (74%) in identifying patients at elevated MACE risk (AUC = 0.78; *p* < 0.001).

**Conclusion:**

Subclinical LVSD, identified by reduced GLS, was strongly associated with adverse cardiovascular events in RA. Whether these findings have therapeutic implications is worth exploring in clinical trials.

**Graphical abstract:**

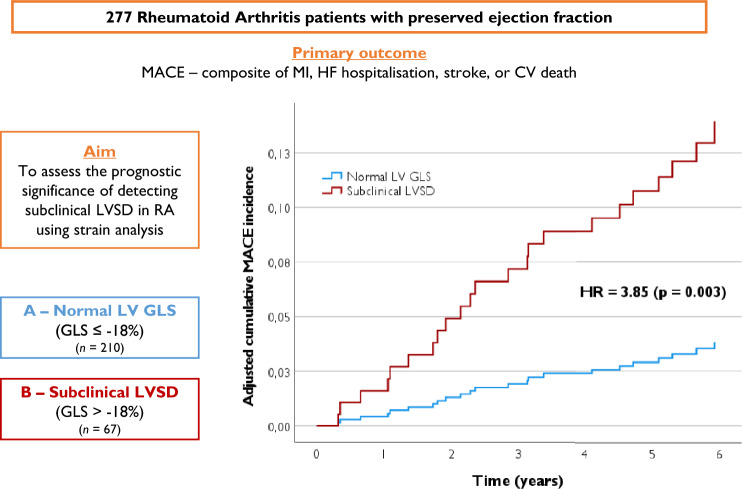

**Supplementary Information:**

The online version contains supplementary material available at 10.1007/s00392-024-02548-6.

## Introduction

Rheumatoid arthritis (RA) is a common rheumatologic disease, affecting approximately 1% of the population [1–4]. It is a systemic disease that has been associated with heart failure (HF) and other cardiac conditions, beyond articular involvement [1, 2]. RA patients have a twofold increased risk of HF, both of ischemic and non-ischemic origin, and mostly with preserved ejection fraction (EF) [1–3, 5, 6]. Traditional cardiovascular risk factors do not explain this increased risk, suggesting that inflammatory pathways may contribute to cardiovascular (CV) disease [2].

In individuals with RA, it is often challenging to ascertain HF symptoms due to functional and musculoskeletal limitation, leading to a significant proportion of patients being underdiagnosed and experiencing poor outcomes [3, 6–8]. Moreover, RA can induce subclinical left ventricular systolic dysfunction (LVSD), which may only manifest as overt left ventricular (LV) dysfunction after an extended preclinical phase [9]. Currently, there are no established guidelines for echocardiographic HF screening specifically tailored to RA patients. Recent research has brought attention to the possibility of expedite detection of subclinical LVSD using echocardiography and surrogate biomarkers [3, 10]. Some studies have already suggested the prognostic utility of N-terminal pro-B-type natriuretic peptide (NT-proBNP), C-reactive protein (CRP), rheumatoid factor (RF), and the echocardiographic assessment of diastolic dysfunction [3, 10, 11]. However, plasma markers like NT-proBNP have been shown to be less effective for CV disease screening in RA, and cardiac involvement may not be detected during a standard echocardiographic examination [6, 12].

Strain analysis has emerged as a promising tool for the early detection of LVSD [13, 14]. LV global longitudinal strain (GLS) can detect subtle changes in myocardial function, often not detectable by EF alone [6, 12]. Thus, LV GLS provides an opportunity for early diagnosis and intervention, with the potential to enhance outcomes and offer valuable long-term prognostic insight.

This study examined a sizable cohort of RA patients without known heart disease. The main aims were: (1) to determine the prevalence of subclinical LVSD detected by reduced LV GLS in RA; (2) to identify predictors of subclinical LVSD in RA patients; and (3) to investigate the prognostic implications of subclinical LVSD detection using strain analysis by assessing its association with the incidence of CV events over a prolonged follow-up period.

## Methods

### Study design and ethical approval

This is a prospective study involving patients from the Autoimmune Disorders outpatient clinic at *Unidade Local de Saúde de Santo António* (Porto, Portugal), who were previously diagnosed with RA and were enrolled between June 2016 and June 2018. The follow-up period extended until September 2023. The protocol for this study was a priori registered at ClinicalTrials.gov under the number NCT03960515.

This study was conducted in accordance with the ethical principles of the 1975 Declaration of Helsinki. The protocol was revised and approved by the Hospital’s Ethics Committee with the number 2016–023(020-DEFI/020-CES). All data were collected for routine clinical purposes and handled anonymously, complying with national norms on data protection. All participants provided written informed consent before enrollment in the study.

### Patient selection

The study cohort comprised individuals diagnosed with RA who fulfilled the 2010 ACR/EULAR Classification Criteria [7], excluding those with a documented history of heart disease. For the present analysis, patients with active neoplasm or severe comorbidity, expected survival of less than 6 months, dementia, inability to walk, or totally dependent in their daily life activities were excluded. Coronary artery disease, past percutaneous or surgical coronary revascularization, previous cardiac surgery, significant valve disease (at least moderate), atrial fibrillation, previously known HF, and reduced EF at the time of the echocardiogram were exclusion criteria. Patients with an inadequate window or poor image quality preventing acceptable strain analysis were also excluded (Fig. S1).

Patients were classified into two categories of LV function for the primary analysis, based on their GLS: Group A—normal LV GLS (≤ − 18%) and Group B—subclinical LVSD (GLS > − 18%). Subclinical LVSD comprised both borderline (− 18% < GLS ≤ − 16%) and abnormal (GLS > − 16%) cases.

### Data collection and variable definitions

#### Clinical data

Biometric and clinical data were collected resourcing to the institution's electronic database. The variables included age, gender, body mass index (BMI), comorbidities, RA disease duration (in years), Disease Activity Score 28 (DAS28), and all medication used (RA and CV medication).

#### Transthoracic echocardiogram

The echocardiographic assessment was conducted by a cardiologist (PR) blinded to the clinical information or outcomes of any other examinations, utilizing a Philips^®^ iE33 ultrasound machine. The assessed parameters included cardiac chamber dimensions (left atrial and end-diastolic ventricular volumes calculated via Simpson’s rule), LV wall thickness and mass, LV EF (determined by the modified Simpson’s biplane method), valvular disease, pericardial disease, and diastolic function as outlined in the 2016 guidelines [15]. LV GLS analysis was performed by a second cardiologist (AA), also blinded to the clinical information and other results, using the vendor-independent software TomTec^®^ (QLAB 13, IntelliSpace Cardiovascular, Philips Medical Systems), according to EACVI/ASE common standards for two-dimensional (2D) speckle-tracking echocardiography [16]. Shortly, 2-chamber, 4-chamber, and long-axis apical cine loops with at least 40 frame rates per second were selected for strain measurement based on 2D speckle-tracking processing. Images were analyzed using a commercial semi-automated software platform (TomTec^®^ QLAB 13). The shortening longitudinal strain of each segment was automatically computed in the three apical views using the 18-segment LV model, and manually adjusted if deemed necessary. When the software couldn’t accurately track the myocardium speckles, it resulted in the exclusion of the corresponding segment. Exclusion of two or more segments determined ineligibility for strain analysis. GLS was determined by averaging the peak systolic strain of all accessible segments.

#### Reproducibility of strain measurements

In our investigation, strain imaging was found to be both feasible and reproducible It was unattainable in only 29 patients (9.5%) due to either an inadequate window or poor image quality. The interobserver reproducibility was assessed using intraclass correlation coefficient: echocardiographic datasets of 28 patients (10% of the studied population) were randomly chosen; a distinct researcher (DSC), blinded to the results of the first observer (AA), reevaluated the same cine imaging loops; the interobserver correlation coefficient for LV GLS was 0.94 (95% CI 0.88–0.97; *p* < 0.001).

#### Biomarkers

Several biomarkers, including NT-proBNP, high-sensitivity troponin T (hsTnT), CRP, and erythrocyte sedimentation rate (ESR), were analyzed. Additionally, the assessment included checking for the presence of anti-cyclic citrullinated peptide (anti-CCP) antibodies or RF and determining the patients’ estimated glomerular filtration rate (eGFR).

#### Main outcomes

The primary outcome was the incidence of major adverse cardiovascular events (MACE), defined as a composite of myocardial infarction (MI), HF hospitalization (any hospitalization lasting more than 24 h due to acute decompensated HF), stroke, or CV death. A key secondary outcome was all-cause mortality, while additional secondary outcomes included 3-Point MACE (3P-MACE, a composite of MI, stroke, or CV death), a composite of HF hospitalization or CV death, CV death alone, and distance covered in 6 min walk test (6MWT).

Outcomes were transformed into annualized event rates by dividing the total number of events by the total person-years of follow-up.

### Statistical analysis

Statistical analysis was performed using the IBM software Statistical Package for Social Sciences (SPSS) version 28.0. Categorical variables were reported by numbers and proportions (%). Continuous variables were presented as means and standard deviations (SD) for normally distributed variables, and medians and interquartile ranges (IQR) for non-normally distributed variables. Comparisons were carried out for each variable using Student’s t test or Mann–Whitney U test for continuous variables, and chi-squared test or Fisher’s exact test for categorical variables, as appropriate. Predictors of subclinical LVSD were determined using Pearson’s correlation coefficients and logistic regression along with the corresponding odds ratios (OR), as appropriate. Survival analyses were performed using the Kaplan–Meier method and Cox proportional hazards models to obtain hazard ratios (HR) and the corresponding 95% confidence intervals (CI). The primary adjustment model included variables identified as clinical predictors of subclinical LVSD: age, hypertension, dyslipidemia, BMI, and eGFR. Adjustment model B was based on the model by Ferreira et al. from the same cohort and included the following covariates: age, sex, diabetes mellitus, RA duration, and eGFR [8]. The discriminative power of LV GLS and LV EF as predictors of MACE was assessed using the Area under the Receiver Operating Characteristic curve (AUC ROC). Covariates with a significant association in the univariate analysis (*p-value* < 0.10) entered the multivariate stage. A two-sided *p-value* < 0.05 was considered to indicate statistical significance.

## Results

### Baseline demographic and clinical characteristics

Of the 399 enrolled patients, a total of 277 were included in this study (Fig. S1). Fig. S2 depicts the distribution of patients across different LV GLS categories. In this cohort, 76% (*n* = 210) exhibited normal LV GLS, whereas 24% (*n* = 67) had subclinical LVSD, including both borderline and abnormal GLS cases.

Detailed characteristics of the patients and echocardiographic parameters are presented in Tables 1 and S1. Mean age was 57 (± 13) years and 79% (*n* = 218) of the participants were women. Patients with subclinical LVSD were older (62 vs 55 years; *p* < 0.001) had higher heart rate (86 vs 78 bpm; *p* < 0.001) and higher body mass index (27.4 vs 25.9 kg/m^2^; *p* = 0.012). Additionally, they were more likely to have dyslipidemia (60% vs 39%; *p* = 0.004) and chronic kidney disease (7% vs 1%; *p* = 0.010). hsTnT was higher in patients with subclinical LVSD (6 vs 4 ng/L; *p* < 0.001) and this group had a lower eGFR (82 vs 93 mL/min/1.73 m^2^; *p* = 0.001). In terms of health-related quality of life indicators, patients with subclinical LVSD covered a shorter distance in the 6MWT (360 vs 390 m; *p* = 0.001).

**Table 1:** Patients’ characteristics across different LV GLS categories

	Overall (*n* = 277)		Normal LV GLS (GLS ≤ − 18%) (*n* = 210)	Subclinical LVSD (GLS > − 18%) (*n* = 67)	*p-value*
Demographic characteristics					
Age (years), mean (± SD)	56.8 (± 12.9)		55.3 (± 13.4)	61.7 (± 10.1)	** < 0.001**
Men, *n* (%)	59 (21.3)		40 (19.0)	19 (28.4)	0.105
Medical history					
Diabetes mellitus, *n* (%)	32 (11.6)		21 (10.0)	11 (16.4)	0.152
HbA1c (%), median (IQR)	5.4 (5.1–5.7)		5.4 (5.1–5.7)	5.5 (5.2–5.9)	**0.005**
Hypertension, *n* (%)	117 (42.2)		82 (39.0)	35 (52.2)	0.057
Smoking, *n* (%)^a^	95 (34.3)		68 (32.4)	27 (40.3)	0.235
Dyslipidemia, *n* (%)	123 (44.4)		83 (39.5)	40 (59.7)	**0.004**
BMI (kg/m^2^), mean (± SD)	26.3 (± 4.2)		25.9 (± 4.1)	27.4 (± 4.1)	**0.012**
Obesity (BMI ≥ 30 kg/m^2^), *n* (%)	47 (17.0)		29 (13.8)	18 (26.9)	**0.013**
Chronic kidney disease, *n* (%)	7 (2.5)		2 (1.0)	5 (7.5)	**0.010**
COPD, *n* (%)	15 (5.4)		9 (4.3)	6 (9.0)	0.210
Family history of IHD, *n* (%)	43 (15.5)		34 (16.2)	9 (13.4)	0.587
Systolic blood pressure (mmHg), median (IQR)	130 (119–142)		128 (117–142)	134 (124–143)	**0.046**
Diastolic blood pressure (mmHg), mean (± SD)	75.7 (± 10.0)		75.3 (± 9.8)	77.0 (± 10.8)	0.225
Heart rate (bpm), mean (± SD)	79.9 (± 13.3)		77.7 (± 12.6)	86.5 (± 13.6)	** < 0.001**
Heart rate > 100 bpm, *n* (%)	21 (7.6)		12 (5.7)	9 (13.4)	**0.038**
RA duration (years), median (IQR)	7.3 (4.1–16.5)		7.2 (4.0–16.1)	9.0 (4.1–18.1)	0.399
DAS28-ESR, mean (± SD)	2.8 (± 1.2)		2.8 (± 1.2)	2.9 (± 1.4)	0.544
Medication					
Cardiovascular medication, *n* (%)					
ACEi or ARB	95 (34.3)		68 (32.4)	27 (40.3)	0.235
Beta-blocker	18 (6.5)		11 (5.2)	7 (10.4)	0.155
Thiazide	44 (15.9)		30 (14.3)	14 (20.9)	0.197
Loop diuretic	2 (0.7)		2 (1.0)	0	1.000
MRA	1 (0.4)		0	1 (1.5)	0.242
CCB	27 (9.7)		20 (9.5)	7 (10.4)	0.824
Statin	94 (33.9)		64 (30.5)	30 (44.8)	**0.031**
RA medication, *n* (%)^b^					
NSAIDs	76 (27.4)		57 (27.1)	19 (28.4)	0.846
Corticosteroids	113 (40.8)		84 (40.0)	29 (43.3)	0.634
Methotrexate	172 (62.1)		134 (63.8)	38 (56.7)	0.297
Biologics	53 (19.1)		39 (18.6)	14 (20.9)	0.674
Other drugs	113 (40.8)		88 (41.9)	25 (37.3)	0.506
Biomarkers					
Biomarkers, median (IQR)					
NT-proBNP (pg/mL) [0–125]	72 (40–128)		75 (39–125)	67 (42–153)	0.559
hsTnT (ng/L) [0–14]	4 (2–7)		4 (2–6)	6 (3–11)	** < 0.001**
ESR (mm) [0–20]	20 (11–35)		20 (11–32)	22 (11–53)	0.213
CRP (mg/L) [0.0–5.0]	2.9 (1.1–7.2)		3.0 (1.2–6.5)	2.8 (1.1–10.8)	0.316
NT-proBNP > 125 pg/mL, *n* (%)	70 (26.0)		51 (25.0)	19 (29.2)	0.498
hsTnT > 14 ng/L, *n* (%)	20 (7.4)		7 (3.4)	13 (20.0)	** < 0.001**
eGFR (mL/min/1.73 m^2^), mean (± SD)	90.4 (± 19.2)		93.2 (± 17.7)	81.6 (± 21.1)	** < 0.001**
eGFR < 60 mL/min/1.73 m^2^, *n* (%)	16 (5.8)		4 (1.9)	12 (17.9)	** < 0.001**
Anti-CCP + or RF + ,* n* (%)	215 (77.6)		164 (78.1)	51 (76.1)	0.735
Health-related quality of life					
Distance in 6MWT (m), median (IQR)	390 (345–435)		390 (360–442)	360 (300–390)	**0.001**
Distance in 6MWT ≤ 440 m,* n* (%)	207 (77.2)		155 (75.2)	52 (83.9)	0.155
HADS-anxiety score, mean (± SD)	6.8 (± 4.5)		6.8 (± 4.3)	6.8 (± 5.0)	0.996
HADS-depression score, mean (± SD)	5.5 (± 4.4)		5.4 (± 4.2)	5.7 (± 4.8)	0.604
Echocardiographic parameters^§^					
Diastolic dysfunction, *n* (%)	36 (13.0)		23 (11.0)	13 (19.4)	0.073
LV EF (%), mean (± SD)	62.0 (± 6.0)		62.9 (± 5.9)	59.2 (± 5.4)	** < 0.001**
LV GLS (%), mean (± SD)	− 20.0 (± 3.2)		− 21.3 (± 2.2)	− 15.8 (± 1.9)	** < 0.001**

Table 1 exhibits patients’ characteristics across different LV GLS categories. Reference values for NT-proBNP, hsTnT, ESR, and CRP are enclosed in square brackets. Significant associations (*p-value* < 0.05) are in bold

^a^Current or previous smoking habits were considered

^b^More than one medication per patient was allowed

^§^Remaining echocardiographic parameters are presented in Table S1

Regarding echocardiography, patients with subclinical LVSD were more likely to have LV hypertrophy (19% vs 3%; *p* < 0.001), increased LV mass index (76.4 vs 64.4 g/m^2^; *p* < 0.001), and relatively lower LV EF (59% vs 63%; *p* < 0.001). With regard to diastolic function, there was a trend toward diastolic dysfunction in patients with subclinical LVSD, although not reaching statistical significance (19% vs 11%; *p* = 0.073). The distribution of LV GLS categories in relation to diastolic function is displayed in Fig. S3.

### Clinical predictors of subclinical LVSD

Table 2 shows clinical predictors of subclinical LVSD. Subclinical LVSD was positively correlated with older age (OR 1.54 per each 10 year increase; *p* < 0.001) and comorbid conditions, such as dyslipidemia (OR 2.27; *p* = 0.004), obesity (OR 2.29; *p* = 0.015), and chronic kidney disease (OR 8.39; *p* = 0.012). Heart rate and hsTnT presented a positive linear correlation (β = 8.7, r = 0.28, *p* < 0.001 for heart rate; and β = 3.6, r = 0.21, *p* < 0.001 for hsTnT), whereas eGFR presented a negative linear correlation with subclinical LVSD (β = -11.6, r = -0.26, *p* < 0.001).

**Table 2:** Clinical predictors of subclinical LVSD (GLS > − 18%)

Clinical predictors of subclinical LVSD (GLS > − 18%)	Univariate predictors			Multivariate predictors		
	OR	95% CI	*p-value*	OR	95% CI	*p-value*
Age (↑ 10 years)	**1.54**	**(1.21–1.97)**	** < 0.001**	1.22	(0.89–1.68)	0.228
Hypertension	1.71	(0.98–2.97)	0.058	0.94	(0.50–1.77)	0.858
HbA1c (↑ 1.0%)	**1.40**	**(1.01–1.92)**	**0.040**	1.29	(0.91–1.82)	0.151
Dyslipidemia	**2.27**	**(1.29–3.97)**	**0.004**	1.62	(0.88–2.97)	0.120
Statin use	**1.85**	**(1.05–3.25)**	**0.033**	1.44	(0.79–2.63)	0.237
BMI (↑ 5 kg/m^2^)	**1.51**	**(1.09–2.09)**	**0.014**	1.37	(0.97–1.93)	0.076
Obesity (BMI ≥ 30 kg/m^2^)	**2.29**	**(1.18–4.47)**	**0.015**	**2.05**	**(1.02–4.11)**	**0.043**
Chronic kidney disease	**8.39**	**(1.59–44.3)**	**0.012**	4.91	(0.88–27.3)	0.069
eGFR (↓ 10 mL/min/1.73 m^2^)	**1.38**	**(1.19–1.62)**	** < 0.001**	**1.24**	**(1.02–1.49)**	**0.029**
eGFR < 60 mL/min/1.73 m^2^	**11.2**	**(3.49–36.2)**	** < 0.001**	**7.25**	**(2.12–24.7)**	**0.002**
Systolic blood pressure (↑ 10 mmHg)	1.10	(0.96–1.28)	0.165	–	–	–
Heart rate (↑ 10 bpm)	**1.63**	**(1.32–2.02)**	** < 0.001**	**1.72**	**(1.37–2.18)**	** < 0.001**
Heart rate > 100 bpm	**2.56**	**(1.03–6.38)**	**0.043**	**2.95**	**(1.10–7.89)**	**0.031**
hsTnT > 14 ng/L	**7.07**	**(2.68–18.6)**	** < 0.001**	**4.36**	**(1.50–12.7)**	**0.007**

Linear Correlations	Unadjusted linear correlations			Adjusted linear correlations		
	β	r	*p-value*	β	R	*p-value*
Age (years)	6.41	0.21	** < 0.001**	–	–	0.453
BMI (kg/m^2^)	1.46	0.15	**0.012**	–	–	0.103
eGFR (mL/min/1.73m^2^)	− 11.6	− 0.26	** < 0.001**	− 4.78	− 0.67	**0.024**
Heart rate (bpm)	8.71	0.28	** < 0.001**	9.27	0.34	** < 0.001**
hsTnT (ng/L)	3.61	0.21	** < 0.001**	1.96	0.49	**0.042**
Log2-hsTnT (ng/L)	0.75	0.29	** < 0.001**	0.38	0.67	**0.002**

Table 2 presents univariate and multivariate clinical predictors of subclinical LVSD (GLS > − 18%), along with the corresponding OR. It also presents linear correlations and the respective Pearson's correlation coefficients (r) between patients’ characteristics (age, BMI, eGFR, heart rate, hsTnT) and subclinical LVSD. The multivariate analysis included the following covariates: age, hypertension, dyslipidemia, BMI, and eGFR. Significant associations (*p-value* < 0.05) are in bold

After adjusting for potential confounding factors, obesity (OR 2.05; *p* = 0.043), eGFR (OR 1.24 per each decrease of 10 mL/min/1.73m^2^; *p* = 0.029), heart rate (OR 1.72 per each 10 bpm increase; *p* < 0.001), and elevated hsTnT (OR 4.36; *p* = 0.007) were multivariate predictors of subclinical LVSD.

### Long-term follow-up and main outcomes

#### Major adverse cardiovascular events (MACE)

The annualized event rates of outcomes by LV GLS categories are found in Table 3, along with adjusted Cox regression. The incidences of outcomes are found in Table S2. During a median follow-up time of 6.1 years (IQR 5.5–6.6), a primary outcome event (MACE) occurred in a total of 23 RA patients (8.3%), comprising 9 patients (4.3%) in group A (normal LV GLS—reference group) and 14 patients (20.9%) in group B (subclinical LVSD).

**Table 3:** Annualized event rates of primary and secondary outcomes categorized by LV GLS

Outcomes	Overall	A—Normal LV GLS (GLS ≤ -18%)	B—Subclinical LVSD (GLS > -18%)	Adjusted Cox regression		
	*Events per 1000 Person-Years*			HR	95% CI	*p-value*
Primary outcome						
MACE	14	7	37	**3.85**	**(1.58–9.37)**	**0.003**
Secondary outcomes						
3P-MACE	9	4	26	**4.98**	**(1.60–15.5)**	**0.005**
HF hosp./CV death	9	4	24	**3.40**	**(1.05–11.0)**	**0.041**
HF hosp.	5	3	13	2.76	(0.67–11.3)	0.159
MI^§^	2	2	3	1.42	(0.12–17.0)	0.782
CV death	4	1	13	7.69	(0.79–74.8)	0.079
All-cause mortality	12	7	26	1.75	(0.63–4.86)	0.281
Distance in 6MWT (m)	390 (345–435)	390 (360–442)	360 (300–390)	1.11*	(0.90–1.35)	0.449

Table 3 displays annualized event rates of primary and secondary outcomes in RA patients, categorized by LV GLS groups, along with adjusted Cox regression and the respective HR. The adjusted Cox regression and the respective HR refer to subclinical LVSD (GLS > -18%) compared to normal LV GLS (GLS ≤ -18%). The adjustment included the following covariates: age, hypertension, dyslipidemia, BMI, and eGFR. MACE consists of a composite of MI, HF hospitalization, stroke, or CV death. 3-Point MACE consists of a composite of MI, stroke, or CV death. Significant associations (*p-value* < 0.05) are in bold

*Odds ratio per each less 50 m walked

**Fig. 1:**
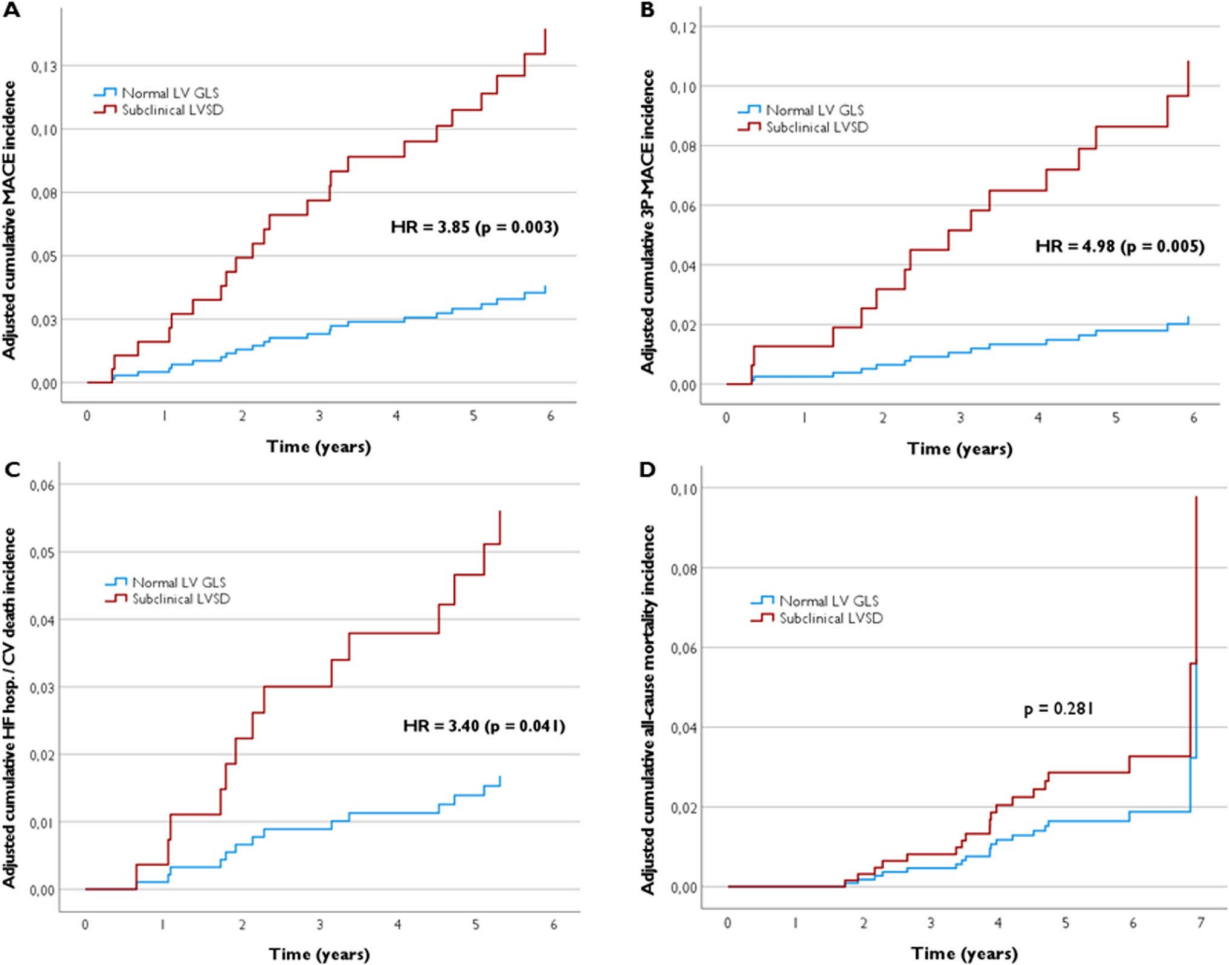
Adjusted cumulative MACE (**A**), 3P-MACE (**B**), HF hospitalization/CV death (**C**), and all-cause mortality (**D**) incidence curves in RA patients, categorized by LV GLS

**Fig. 2:**
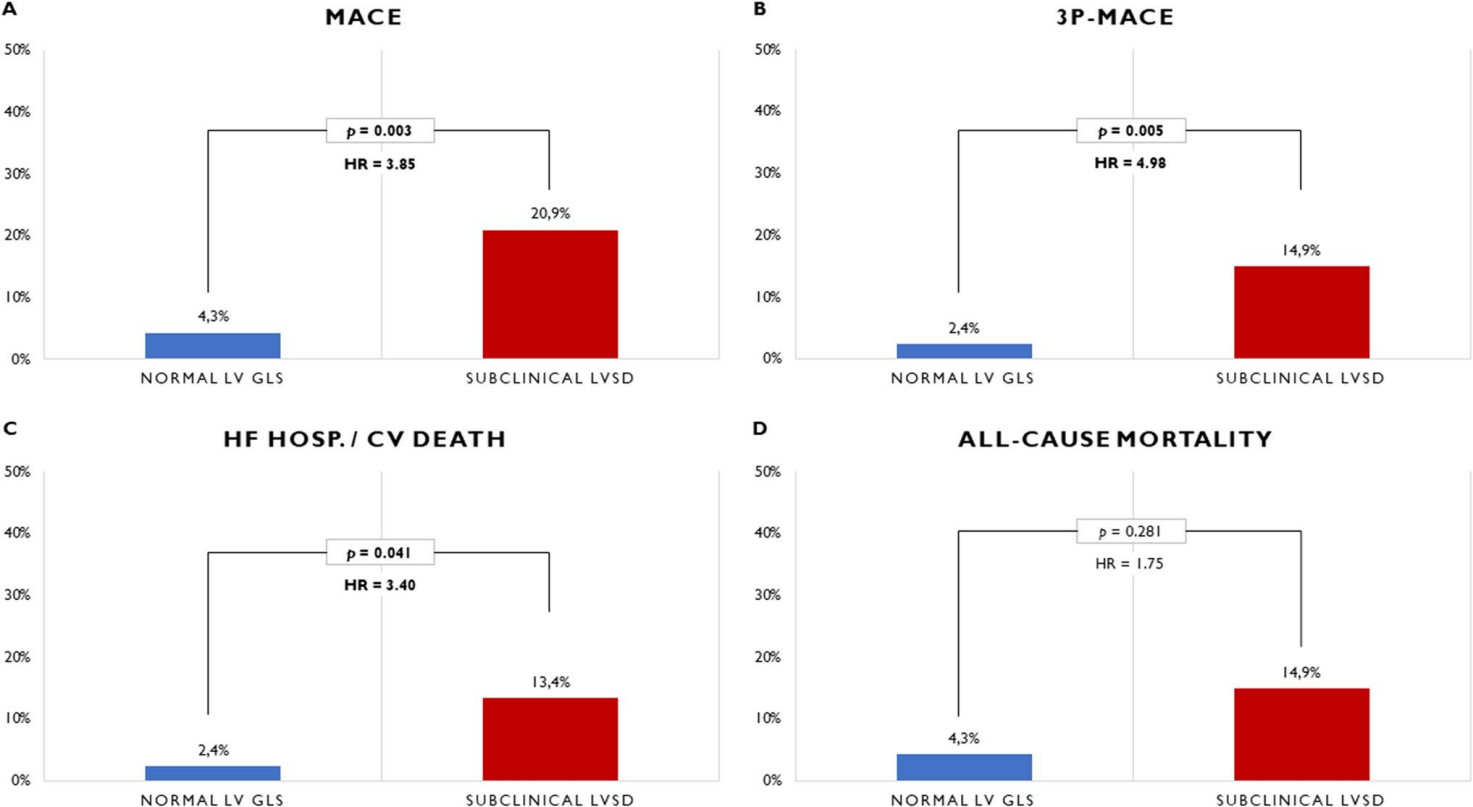
Incidence of MACE (**A**), 3P-MACE (**B**), HF hospitalization/CV death (**C**), and all-cause mortality (**D**) in RA patients, categorized by LV GLS

In adjusted analysis, subclinical LVSD was independently associated with a fourfold higher risk of MACE (HR 3.85; 95% CI 1.58–9.37; *p* = 0.003) compared to patients with normal LV GLS (see Table 3). Figures 1 and 2 show the adjusted cumulative incidence curves and the respective incidences of main outcomes. Additionally, the impact of LV GLS on MACE is demonstrated through Kaplan–Meier survival analysis in Fig. S4. Table S3 presents another model of adjusted (Model B) Cox regression analysis, indicating consistent findings.

In terms of diastolic function, there were no significant differences in the occurrence of MACE between patients with normal diastolic function and those with indeterminate or diastolic dysfunction (*p* = 0.215 and *p* = 0.517, respectively—refer to Table S4). The cumulative MACE-free survival curves in RA patients, categorized by diastolic function, are illustrated in Fig. S5.

#### Secondary outcomes

The findings regarding secondary outcomes are detailed in Table 3. Subclinical LVSD was independently associated with a fivefold higher risk of 3P-MACE (HR 4.98; 95% CI 1.60–15.5; *p* = 0.005) and a threefold higher risk of HF hospitalization or CV death (HR 3.40; 95% CI 1.05–11.0; *p* = 0.041). In terms of all-cause mortality, no significant differences were observed after adjusting for potential confounding factors (HR 1.75; 95% CI 0.63–4.86; *p* = 0.28). Regarding distance in 6MWT, the differences were attenuated after adjustment (OR 1.11 per each less 50 m walked; 95% CI 0.90–1.35; *p* = 0.45). Figures 1 and 2 provide a comprehensive overview of the incidence of secondary outcomes (3P-MACE, HF hosp./CV death, and all-cause mortality).

### ROC analysis

**Fig. 3:**
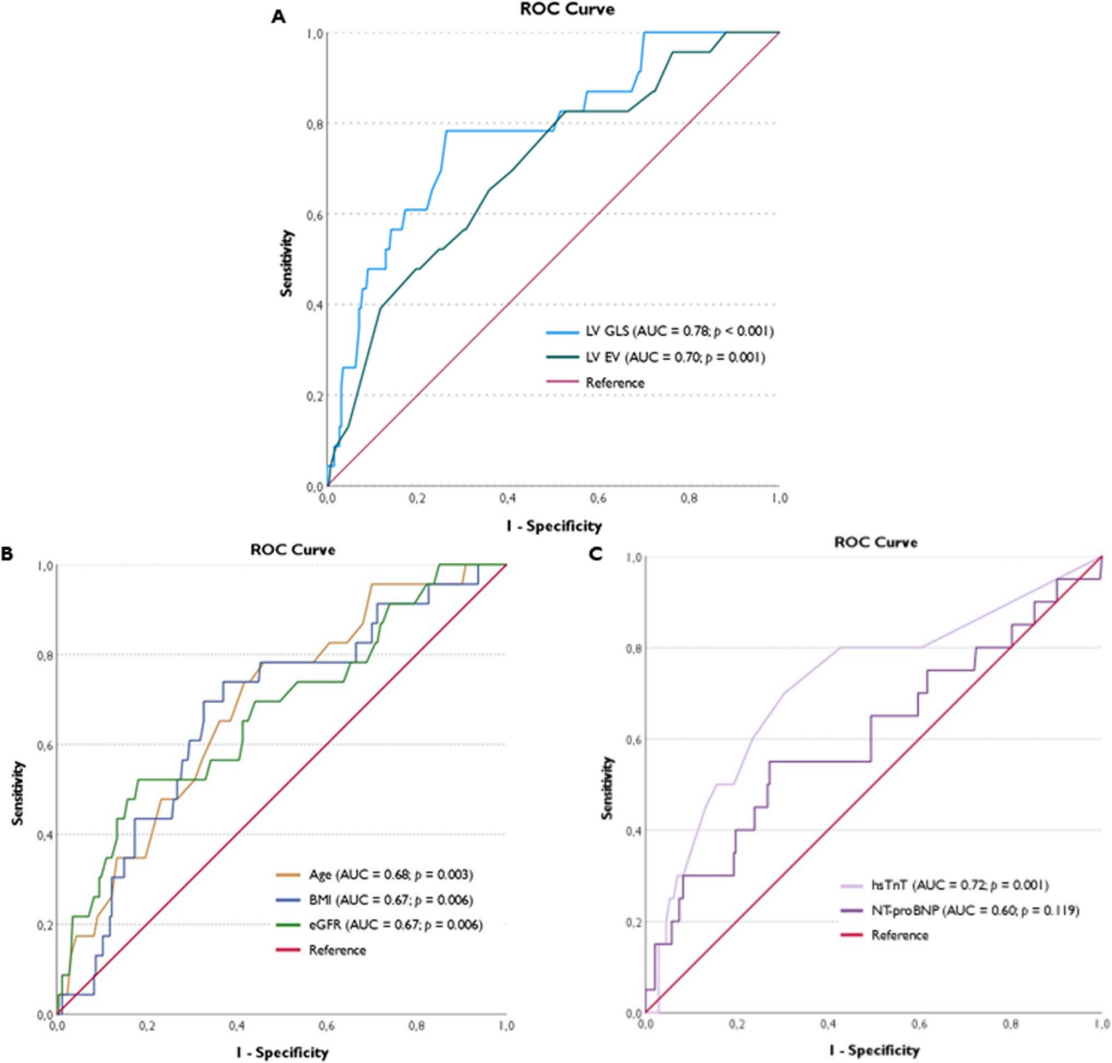
ROC analysis comparing predictive power of echocardiographic parameters (LV GLS and LV EF) [A], HF with preserved EF-related factors (**B**), and biomarkers (**C**) for the occurrence of MACE in RA patients with preserved LV EF

The ROC analysis assessing the predictive power of LV GLS and LV EF for the occurrence of MACE indicated that LV GLS (AUC = 0.78; 95% CI 0.68–0.88; *p* < 0.001) outperformed LV EF (AUC = 0.70; 95% CI 0.59–0.81; *p* = 0.001) as a more sensitive and specific predictor of MACE (Fig. 3A). A LV GLS threshold of > − 18.5% provided optimal sensitivity (78%) and specificity (74%) in identifying RA patients at elevated MACE risk. Figure 3B and C shows two additional ROC analyses comparing the predictive power of different factors, such as age, BMI, eGFR, and biomarkers like hsTnT and NT-proBNP, for the occurrence of MACE. Theses ROC analyses enhanced the understanding of the interplay between these factors and their contribution to CV outcomes in RA patients, while demonstrating that LV GLS remained a more sensitive and specific predictor of MACE than all other variables.

## Discussion

This study represents the longest prospective outcome investigation of subclinical LVSD detected by speckle-tracking echocardiography in RA patients with preserved LV EF and without overt cardiac disease. Key findings include: 1) nearly one-fourth (24%) of RA patients exhibit subclinical LVSD; 2) these patients face a fourfold increased risk of MACE; 3) subclinical LVSD independently predicts MACE in the mid- and long-term follow-up.

In 2021, Ferreira et al. investigated the prevalence, clinical risk factors, and proteomic biomarkers associated with HF development in this same cohort of RA patients, concluding that classical CV risk factors, RA duration, and age are associated with HF risk [8]. Additionally, the proteomic profile provided insights into some of the mechanistic pathways for HF development in these patients [8]. The reported incidence of CV events (composite of CV death and CV hospitalization) during the follow-up period was 10% [8]. HF development was associated, as expected, with a higher risk of CV events (HR 2.37; 95% CI 1.07–5.30; *p* = 0.034) during a median follow-up of four years [8].

Also in 2021, Rodrigues et al. studied the prevalence of ventricular systolic (evaluated by LV EF) and diastolic dysfunction, aiming to identify the predictors of ventricular dysfunction in RA patients, as well as analyzing its associations with surrogate prognostic markers [3]. The study concluded that RA patients without overt cardiac disease had a prevalence of systolic and diastolic dysfunction of 4% and 13%, respectively, with age being associated with a higher prevalence of ventricular dysfunction [3]. Notably, this study compared RA patients with a cohort from the general population of the same city, revealing a higher prevalence of diastolic dysfunction among RA patients (13% vs 1.4%; *p* < 0.001) [3].

The present study expands the knowledge about this reasonably large RA cohort, particularly by adding information about subclinical LVSD using LV GLS as an early sign of LVSD, thereby increasing the sensitivity of its detection. Furthermore, the longer follow-up period allowed by the time span between these analyses permitted the observation of a larger number of CV events, offering a more detailed description of the natural history of CV disease in RA patients.

Our study’s findings align with earlier reports on reduced LV strain in RA patients without overt cardiac disease [1, 4–6, 17]. The hypothesis that impaired ventricular strain serves as an early and subclinical marker for CV disease has been gaining support. Previous studies have noted decreased global LV strain in RA patients compared to normal individuals [9, 12, 18]. For instance, Cioffi et al. demonstrated that a low LV GLS was detected in 24% of RA patients and in only 5% of matched control individuals [9, 18]. However, most of these studies involved a smaller or comparable number of patients and did not investigate the prognostic role of subclinical LVSD through long-term follow-up.

Our larger-scale research with an extended follow-up period strengthens the evidence that LV strain measurement can serve as a sensitive indicator of early myocardial dysfunction in RA. In our study, nearly one-fourth of RA patients exhibited subclinical LVSD, with one-tenth showing abnormal LV GLS. Although the prevalence of abnormal GLS in our cohort was lower than reported in some previous RA studies, such as the 24% prevalence reported by Cioffi et al. [18], our findings reveal a higher proportion of abnormal LV GLS (10%) compared to that observed in a normal matched control group (5%) [18]. The lower proportion of abnormal GLS cases observed in our study could be attributed to more stringent exclusion criteria, particularly excluding patients with severe comorbidities, as well as the exclusion of individuals with reduced LV EF. Still, the prevalence of LVSD as assessed by GLS impairment was nearly twofold higher than that detected in patients used as matched control representing the general health population [18]. These findings underscore the presence of subclinical disease in RA patients despite a normal LV EF and highlight the efficacy of myocardial strain imaging in its detection [1, 12].

Over a median follow-up of 6.1 years, MACE events were observed in 8% of all RA patients and in 21% of those with subclinical LVSD. The yearly all-cause mortality was approximately 1.1%, which is lower than the mortality rates reported in other studies [10, 19]. This could be attributed to our study's exclusion criteria, which excluded critically ill patients with RA, those with an expected survival of less than 6 months, active neoplasm, or previously known heart disease. Notably, subclinical LVSD was independently associated with a fourfold higher risk of MACE, a fivefold higher risk of 3P-MACE, and a threefold higher risk of HF hospitalization or CV death compared to patients with normal LV GLS. Concerning other secondary outcomes, no significant differences were observed in all-cause mortality and distance in 6MWT after adjusting for potential confounding factors.

The notably increased incidence of MACE among patients with reduced LV GLS, compared to those with normal strain, within this highly selected cohort emphasizes the prognostic significance of this imaging marker in identifying patients at heightened risk. Additionally, the comparison between LV GLS and LV EF as predictors of MACE indicated that LV GLS outperformed LV EF in terms of sensitivity and specificity. The ROC analysis highlighted a LV GLS threshold of > − 18.5% as optimal for identifying RA patients at elevated risk of MACE, with a sensitivity 78% and a specificity of 74%. This analysis further demonstrated that LV GLS remained a more accurate predictor of MACE compared to all other variables.

Identifying predictors of subclinical LVSD is crucial for effective screening. In our analysis, patients with reduced LV GLS were older, exhibited higher prevalence of dyslipidemia and chronic kidney disease, higher body mass index, heart rate, and hsTnT levels. After adjusting for potential confounding factors, obesity, lower eGFR, higher heart rate, and elevated hsTnT emerged as multivariate predictors of subclinical LVSD. This indicates potential screening benefits for obese and chronic kidney disease patients. Concerning heart rate, we observed a positive linear correlation with subclinical LVSD, with an adjusted OR of 1.72 per each 10 bpm increase, aligning with previous research [20]. However, the therapeutic implications of this finding require further exploration in clinical trials, since the benefit of beta-blockers in HF with preserved EF remains uncertain [21, 22]. Regarding hsTnT, we observed an association between elevated hsTnT levels (> 14 ng/L) and subclinical LVSD, with an adjusted OR of 4.36. In contrast, no similar association was found with NT-proBNP, suggesting hsTnT’s potential as a more sensitive and earlier biomarker of cardiac dysfunction in RA.

Our cohort exhibited a substantial prevalence of traditional CV risk factors, including diabetes, dyslipidemia, and hypertension, especially in the subclinical LSVD group. However, the prevalence of diabetes in our cohort was comparable to that of the general Portuguese population, which was estimated to be 11.7% in 2010 and has since increased to 13%, according to the International Diabetes Federation [23, 24]. The association between RA and an increased risk of CV disease is well-established, with RA patients experiencing an estimated 1.5 times higher risk [25, 26]. This risk burden is multifactorial, stemming from the higher prevalence of traditional risk factors as well as chronic inflammation [27]. Inflammation has a direct effect on endothelial cells and atherosclerotic disease progression, independently contributing to CV disease [28, 29]. The cumulative effect of these classical risk factors and chronic inflammation is likely significant in this subgroup. Nevertheless, our study demonstrated that subclinical LVSD remained independently associated with a higher risk of MACE.

Compared to the general population, the annualized event rates for MACE and all-cause mortality were higher in our study cohort. A study by Yusuf et al. found that in a broadly comparable population in high-income countries, the incidences of MACE and all-cause mortality were lower than in our population (4 vs 14 and 2 vs 12 events per 1000 person-years, respectively) [30]. When compared to a diabetic population in a large population-based study, our cohort showed a comparable MACE risk (14 events per 1000 person-years) [31]. Regarding chronic kidney disease, the MACE risk in our RA population was lower compared to a large population-based study with mild to moderate chronic kidney disease (14 vs 36.5 events per 1000 person-years), while all-cause mortality was comparable (12 vs 11 events per 1000 person-years) [32]. However, in the later study, MACE included peripheral artery disease events, which were not captured in our study population, potentially explaining the higher event rates reported. Therefore, although RA is known to increase global CV and mortality risks, our carefully selected population likely excluded many of the higher-risk patients, resulting in an overall low-to-intermediate risk population.

Some studies have linked high disease activity to CV disease in RA, irrespective of traditional risk factors [33, 34]. However, our study found no association between RA activity characteristics (e.g., RA duration, DAS28-ESR score) or inflammatory markers (e.g., ESR and CRP) and subclinical LVSD, underscoring the challenges of using these biomarkers in early cardiac disease detection.

In terms of other echocardiographic characteristics, while patients with subclinical LVSD showed a trend toward diastolic dysfunction, statistical significance was not reached. Additionally, no significant differences in MACE incidence were found between patients with various diastolic function statuses. This underscores LV GLS's potential as a sensitive tool for early myocardial dysfunction detection, independent of diastolic dysfunction.

Our findings support the hypothesis that impaired ventricular strain could serve as an early marker for CV disease, prompting further investigations into targeted interventions and management strategies. However, the integration of these results into clinical practice remains unclear. Studies by Shah et al. [35] and Minamisawa et al. [36] suggest potential benefits with spironolactone but inconclusive results with sacubitril/valsartan in improving GLS in a population with HF with preserved EF, emphasizing the need for additional research in clinical trials and specifically in RA patients.

### Strengths and limitations

This study has certain limitations that should be considered. First, the lack of a sex- and gender-matched healthy control group hinders direct comparisons and precludes conclusions about the specific impact of RA on subclinical LVSD. Second, while the inclusion and exclusion criteria ensure a more selective cohort and enhance the internal validity of our study, they also limit the generalizability and comparability to existing literature. Third, despite the prospective nature of the study, the observational design prevents causal inference. Fourth, although the relatively long follow-up period is a strength, providing valuable insights into the natural history of the disease and allowing for the capture of more clinical events, it also imposes limitations on the interpretation of results, particularly due to the lack of information on treatment changes over time. Additionally, the absence of systematic follow-up echocardiography data, due to resource constraints and logistical challenges, represents another limitation that future research could address. Lastly, the effectiveness of interventions targeting subclinical LVSD on clinical outcomes remains uncertain, reflecting a gap in the current knowledge for this subpopulation. Notwithstanding these limitations, the prospective design and the inclusion of a relatively large and thoroughly selected sample of RA patients make this study one of the most comprehensive descriptions of the natural history of cardiac disease in RA patients.

## Conclusions

LV GLS is a valuable tool for detecting subclinical LVSD in RA patients with preserved EF. Subclinical LVSD, defined as LV GLS > − 18%, significantly increases the risk of MACE by fourfold compared to individuals with normal LV GLS. Whether these findings have therapeutic implication is worth exploring in clinical trials.

## Supplementary Information

Below is the link to the electronic supplementary material.

Supplementary file1 (TIF 914 KB)

Supplementary file2 (TIF 382 KB)

Supplementary file3 (TIF 909 KB)

Supplementary file4 (TIF 867 KB)

Supplementary file5 (TIF 1034 KB)

Supplementary file6 (DOCX 1034 KB)

## Data Availability

The data that support the findings of this study are available from the corresponding author upon reasonable request.

## References

[1] Thallapally VK, Bansal R, Thandra A, Gupta S, Aurit S, Pajjuru VS et al (2023) Detection of myocardial dysfunction using global longitudinal strain with speckle-tracking echocardiography in patients with vs without rheumatoid arthritis: a systematic review and meta-analysis. J Echocardiogr 21(1):23–32. 10.1007/s12574-022-00583-8 35987937 10.1007/s12574-022-00583-8

[2] Solomon DH, Karlson EW, Rimm EB, Cannuscio CC, Mandl LA, Manson JE et al (2003) Cardiovascular morbidity and mortality in women diagnosed with rheumatoid arthritis. Circulation 107(9):1303–1307. 10.1161/01.cir.0000054612.26458.b2 12628952 10.1161/01.cir.0000054612.26458.b2

[3] Rodrigues P, Ferreira B, Fonseca T, Costa RQ, Cabral S, Pinto JL et al (2021) Subclinical ventricular dysfunction in rheumatoid arthritis. Int J Cardiovasc Imaging 37(3):847–859. 10.1007/s10554-020-02057-3 33052554 10.1007/s10554-020-02057-3

[4] Sitia S, Tomasoni L, Cicala S, Atzeni F, Ricci C, Gaeta M et al (2012) Detection of preclinical impairment of myocardial function in rheumatoid arthritis patients with short disease duration by speckle tracking echocardiography. Int J Cardiol 160(1):8–14. 10.1016/j.ijcard.2011.03.012 21450355 10.1016/j.ijcard.2011.03.012

[5] Naseem M, Samir S, Ibrahim IK, Khedr L, Shahba AAE (2019) 2-D speckle-tracking assessment of left and right ventricular function in rheumatoid arthritis patients with and without disease activity. J Saudi Heart Assoc 31(1):41–49. 10.1016/j.jsha.2018.10.001 30559579 10.1016/j.jsha.2018.10.001 PMC6289904

[6] Baktir AO, Sarli B, Cebicci MA, Saglam H, Dogan Y, Demirbas M et al (2015) Preclinical impairment of myocardial function in rheumatoid arthritis patients. Detection of myocardial strain by speckle tracking echocardiography. Herz 40(4):669–674. 10.1007/s00059-014-4068-3 24595319 10.1007/s00059-014-4068-3

[7] Villeneuve E, Nam J, Emery P (2010) 2010 ACR-EULAR classification criteria for rheumatoid arthritis. Rev Bras Reumatol 50(5):481–483 21125184

[8] Ferreira MB, Fonseca T, Costa R, Marinhoc A, Carvalho HC, Oliveira JC et al (2021) Prevalence, risk factors and proteomic bioprofiles associated with heart failure in rheumatoid arthritis: the RA-HF study. Eur J Intern Med 85:41–49. 10.1016/j.ejim.2020.11.002 33162300 10.1016/j.ejim.2020.11.002

[9] Cioffi G, Giollo A, Orsolini G, Idolazzi L, Carletto A, Ognibeni F et al (2020) Incidence and predictors of adverse clinical events in patients with rheumatoid arthritis and asymptomatic left ventricular systolic dysfunction. Clin Exp Rheumatol 38(3):420–427 31577214

[10] Provan S, Angel K, Semb AG, Atar D, Kvien TK (2010) NT-proBNP predicts mortality in patients with rheumatoid arthritis: results from 10 year follow-up of the EURIDISS study. Ann Rheum Dis 69(11):1946–1950. 10.1136/ard.2009.127704 20525846 10.1136/ard.2009.127704

[11] Aslam F, Bandeali SJ, Khan NA, Alam M (2013) Diastolic dysfunction in rheumatoid arthritis: a meta-analysis and systematic review. Arthritis Care Res (Hoboken) 65(4):534–543. 10.1002/acr.21861 23002032 10.1002/acr.21861

[12] Fine NM, Crowson CS, Lin G, Oh JK, Villarraga HR, Gabriel SE (2014) Evaluation of myocardial function in patients with rheumatoid arthritis using strain imaging by speckle-tracking echocardiography. Ann Rheum Dis 73(10):1833–1839. 10.1136/annrheumdis-2013-203314 23873875 10.1136/annrheumdis-2013-203314 PMC3895498

[13] Hanvivadhanakul P, Buakhamsri A (2019) Disease activity is associated with LV dysfunction in rheumatoid arthritis patients without clinical cardiovascular disease. Adv Rheumatol 59(1):56. 10.1186/s42358-019-0100-x 31843000 10.1186/s42358-019-0100-x

[14] Thavendiranathan P, Poulin F, Lim KD, Plana JC, Woo A, Marwick TH (2014) Use of myocardial strain imaging by echocardiography for the early detection of cardiotoxicity in patients during and after cancer chemotherapy: a systematic review. J Am Coll Cardiol 63(25):2751–2768. 10.1016/j.jacc.2014.01.073 24703918 10.1016/j.jacc.2014.01.073

[15] Nagueh SF, Smiseth OA, Appleton CP, Byrd BF 3rd, Dokainish H, Edvardsen T et al (2016) Recommendations for the evaluation of left ventricular diastolic function by echocardiography: an update from the American society of echocardiography and the European association of cardiovascular imaging. Eur Heart J Cardiovasc Imaging 17(12):1321–1360. 10.1093/ehjci/jew082 27422899 10.1093/ehjci/jew082

[16] Voigt JU, Pedrizzetti G, Lysyansky P, Marwick TH, Houle H, Baumann R et al (2015) Definitions for a common standard for 2D speckle tracking echocardiography: consensus document of the EACVI/ASE/industry task force to standardize deformation imaging. Eur Heart J Cardiovasc Imaging 16(1):1–11. 10.1093/ehjci/jeu184 25525063 10.1093/ehjci/jeu184

[17] Benacka O, Benacka J, Blazicek P, Belansky M, Payer J, Killinger Z et al (2017) Speckle tracking can detect subclinical myocardial dysfunction in rheumatoid arthritis patients. Bratisl Lek Listy 118(1):28–33. 10.4149/BLL_2017_006 28127980 10.4149/BLL_2017_006

[18] Cioffi G, Viapiana O, Ognibeni F, Dalbeni A, Giollo A, Gatti D et al (2017) Prognostic role of subclinical left ventricular systolic dysfunction evaluated by speckle-tracking echocardiography in rheumatoid arthritis. J Am Soc Echocardiogr 30(6):602–611. 10.1016/j.echo.2017.02.001 28391000 10.1016/j.echo.2017.02.001

[19] Gonzalez A, Maradit Kremers H, Crowson CS, Nicola PJ, Davis JM 3rd, Therneau TM et al (2007) The widening mortality gap between rheumatoid arthritis patients and the general population. Arthritis Rheum 56(11):3583–3587. 10.1002/art.22979 17968923 10.1002/art.22979

[20] Nyberg J, Jakobsen EO, Ostvik A, Holte E, Stolen S, Lovstakken L et al (2023) Echocardiographic reference ranges of global longitudinal strain for all cardiac chambers using guideline-directed dedicated views. JACC Cardiovasc Imaging 16(12):1516–1531. 10.1016/j.jcmg.2023.08.011 37921718 10.1016/j.jcmg.2023.08.011

[21] Peikert A, Bart BA, Vaduganathan M, Claggett BL, Kulac IJ, Kosiborod MN et al (2023) Contemporary use and implications of beta-blockers in patients With HFmrEF or HFpEF: the deliver trial. JACC Heart Fail. 10.1016/j.jchf.2023.09.007 37767674 10.1016/j.jchf.2023.09.007

[22] Kaddoura R, Madurasinghe V, Chapra A, Abushanab D, Al-Badriyeh D, Patel A (2024) Beta-blocker therapy in heart failure with preserved ejection fraction (B-HFpEF): a systematic review and meta-analysis. Curr Probl Cardiol 49(3):102376. 10.1016/j.cpcardiol.2024.102376 38184132 10.1016/j.cpcardiol.2024.102376

[23] Gardete-Correia L, Boavida JM, Raposo JF, Mesquita AC, Fona C, Carvalho R et al (2010) First diabetes prevalence study in Portugal: PREVADIAB study. Diabet Med 27(8):879–881. 10.1111/j.1464-5491.2010.03017.x 20653744 10.1111/j.1464-5491.2010.03017.x

[24] International Diabetes Federation: Diabetes in Portugal (2021). https://idf.org/europe/our-network/our-members/portugal/ (2021). Accessed 10 Aug 2024

[25] Duruöz MT, Ataman Ş, Bodur H, Çay HF, Melikoğlu MA, Akgül Ö et al (2024) Prevalence of cardiovascular diseases and traditional cardiovascular risk factors in patients with rheumatoid arthritis: a real-life evidence from BioSTAR nationwide registry. Rheumatol Int 44(2):291–301. 10.1007/s00296-023-05515-y 38157014 10.1007/s00296-023-05515-y

[26] Dijkshoorn B, Raadsen R, Nurmohamed MT (2022) Cardiovascular disease risk in rheumatoid arthritis anno 2022. J Clin Med. 10.3390/jcm11102704 35628831 10.3390/jcm11102704 PMC9142998

[27] Jagpal A, Navarro-Millan I (2018) Cardiovascular co-morbidity in patients with rheumatoid arthritis: a narrative review of risk factors, cardiovascular risk assessment and treatment. BMC Rheumatol 2:10. 10.1186/s41927-018-0014-y 30886961 10.1186/s41927-018-0014-y PMC6390616

[28] van Halm VP, Peters MJ, Voskuyl AE, Boers M, Lems WF, Visser M et al (2009) Rheumatoid arthritis versus diabetes as a risk factor for cardiovascular disease: a cross-sectional study, the CARRE Investigation. Ann Rheum Dis 68(9):1395–1400. 10.1136/ard.2008.094151 18697775 10.1136/ard.2008.094151

[29] Liao KP (2017) Cardiovascular disease in patients with rheumatoid arthritis. Trends Cardiovasc Med 27(2):136–140. 10.1016/j.tcm.2016.07.006 27612551 10.1016/j.tcm.2016.07.006 PMC5253086

[30] Yusuf S, Rangarajan S, Teo K, Islam S, Li W, Liu L et al (2014) Cardiovascular risk and events in 17 low-, middle-, and high-income countries. N Engl J Med 371(9):818–827. 10.1056/NEJMoa1311890 25162888 10.1056/NEJMoa1311890

[31] Ke C, Lipscombe LL, Weisman A, Zhou L, Austin PC, Shah BR et al (2022) Trends in the association between diabetes and cardiovascular events, 1994–2019. JAMA 328(18):1866–1869. 10.1001/jama.2022.14914 36239969 10.1001/jama.2022.14914 PMC9568883

[32] Go AS, Chertow GM, Fan D, McCulloch CE, Hsu CY (2004) Chronic kidney disease and the risks of death, cardiovascular events, and hospitalization. N Engl J Med 351(13):1296–1305. 10.1056/NEJMoa041031 15385656 10.1056/NEJMoa041031

[33] Midtbo H, Semb AG, Matre K, Kvien TK, Gerdts E (2017) Disease activity is associated with reduced left ventricular systolic myocardial function in patients with rheumatoid arthritis. Ann Rheum Dis 76(2):371–376. 10.1136/annrheumdis-2016-209223 27269296 10.1136/annrheumdis-2016-209223

[34] Maradit-Kremers H, Nicola PJ, Crowson CS, Ballman KV, Gabriel SE (2005) Cardiovascular death in rheumatoid arthritis: a population-based study. Arthritis Rheum 52(3):722–732. 10.1002/art.20878 15751097 10.1002/art.20878

[35] Shah AM, Claggett B, Sweitzer NK, Shah SJ, Anand IS, Liu L et al (2015) Prognostic importance of impaired systolic function in heart failure with preserved ejection fraction and the impact of spironolactone. Circulation 132(5):402–414. 10.1161/CIRCULATIONAHA.115.015884 26130119 10.1161/CIRCULATIONAHA.115.015884 PMC4526442

[36] Minamisawa M, Inciardi RM, Claggett B, Cikes M, Liu L, Prasad N et al (2024) Clinical implications of subclinical left ventricular dysfunction in heart failure with preserved ejection fraction: the PARAGON-HF study. Eur J Heart Fail. 10.1002/ejhf.3167 38369856 10.1002/ejhf.3167

