# Supplementary material for: Subclinical left ventricular dysfunction in rheumatoid arthritis: findings from the prospective Porto-RA cohort
Source: Clin Res Cardiol. 2024 Sep 30;114(9):1191–203. doi: 10.1007/s00392-024-02548-6 (PMC12408714; doi:10.1007/s00392-024-02548-6)
Supplement: Supplementary file 6 — Supplementary file6 (DOCX 1034 KB) [file 392_2024_2548_MOESM6_ESM.docx]

**Supplementary Files**

**Supplementary Tables**

**Table S1: Echocardiographic characteristics of the study population.**

| **Echocardiographic parameters** | **Overall**  (*n*=277) | **Normal LV GLS**  (GLS ≤ -18%)  (*n*=210) | **Subclinical LVSD**  (GLS > -18%)  (*n*=67) | ***p-value*** |
| --- | --- | --- | --- | --- |
| LV end-diastolic diameter index (mm/m^2^), median (IQR) | 26 (24-27) | 26 (24-27) | 25 (23-27) | **0.023** |
| LV end-systolic diameter index (mm/m^2^), median (IQR) | 16 (14-19) | 16 (14-19) | 16 (14-18) | 0.625 |
| LV end-diastolic volume index (mL/m^2^), median (IQR) | 47 (40-54) | 47 (41-54) | 44 (38-53) | 0.057 |
| Stroke volume index (mL/m^2^), median (IQR) | 38 (32-44) | 38 (32-44) | 36 (31-44) | 0.205 |
| LV relative wall thickness, median (IQR) | 0.36 (0.30-0.43) | 0.35 (0.30-0.42) | 0.41 (0.35-0.50) | **<0.001** |
| LV mass index (g/m^2^), mean (±SD) | 67.3 (±18.9) | 64.4 (±18.1) | 76.4 (±18.8) | **<0.001** |
| LV hypertrophy, *n* (%) | 20 (7.2) | 7 (3.3) | 13 (19.4) | **<0.001** |
| Left atrial volume index (mL/m^2^), mean (±SD) | 31.5 (±9.1) | 31.8 (±9.3) | 30.4 (±8.6) | 0.281 |
| Peak E wave velocity (cm/s), median (IQR) | 70 (61-82) | 72 (63-85) | 64 (58-76) | **0.003** |
| Deceleration time (ms), median (IQR) | 147 (120-180) | 150 (120-180) | 130 (100-190) | 0.089 |
| E/A ratio, median (IQR) | 0.93 (0.77-1.20) | 0.97 (0.80-1.30) | 0.84 (0.66-1.00) | **<0.001** |
| E/e’ ratio, median (IQR) | 8 (7-10) | 8 (7-10) | 9 (8-12) | **0.004** |
| Diastolic dysfunction, *n* (%) | 36 (13.0) | 23 (11.0) | 13 (19.4) | 0.073 |
| TAPSE (mm), median (IQR) | 24 (21-26) | 24 (22-27) | 23 (20-25) | **0.003** |
| Estimated PASP (mmHg), median (IQR) | 23 (19-26) | 23 (19-26) | 22 (19-26) | 0.793 |
| LV EF (%), mean (±SD) | 62.0 (±6.0) | 62.9 (±5.9) | 59.2 (±5.4) | **<0.001** |
| LV GLS (%), mean (±SD) | -20.0 (±3.2) | -21.3 (±2.2) | -15.8 (±1.9) | **<0.001** |

Table S1 exhibits echocardiographic characteristics of the study population across different LV GLS categories. Significant associations (*p-value* < 0.05) are in bold.

Abbreviations: EF: ejection fraction; GLS: global longitudinal strain; IQR: interquartile range; LV: left ventricle/cular; PASP: pulmonary artery systolic pressure; SD: standard deviation; TAPSE: tricuspid annular plane systolic excursion.

**Table S2: Primary and secondary outcomes categorised by LV GLS.**

| **Outcomes** | **Overall**  (*n*=277) | **A - Normal LV GLS**  (GLS ≤ -18%)  (*n*=210) | **B - Subclinical LVSD**  (GLS > -18%)  (*n*=67) |
| --- | --- | --- | --- |
|  | *number of patients (percent)* | | |
| **Primary outcome** |  |  |  |
| MACE | 23 (8.3) | 9 (4.3) | 14 (20.9) |
| **Secondary outcomes** |  |  |  |
| 3P-MACE | 15 (5.4) | 5 (2.4) | 10 (14.9) |
| HF hosp. / CV death | 14 (5.1) | 5 (2.4) | 9 (13.4) |
| HF hosp. | 9 (3.2) | 4 (1.9) | 5 (7.5) |
| MI^§^ | 3 (1.1) | 2 (1.0) | 1 (1.5) |
| CV death | 6 (2.2) | 1 (0.5) | 5 (7.5) |
| All-cause mortality | 19 (6.9) | 9 (4.3) | 10 (14.9) |

Table S2 displays the incidence of primary and secondary outcomes in RA patients, categorised by LV GLS groups.

MACE consists of a composite of MI, HF hospitalisation, stroke, or CV death.

3-Point MACE consists of a composite of MI, stroke, or CV death.

^§^Regarding MI, group A included one ST-elevation myocardial infarction and one non-ST-elevation myocardial infarction, while group B included one non-ST-elevation myocardial infarction.

Significant associations (*p-value* < 0.05) are in bold.

Abbreviations: CV: cardiovascular; GLS: global longitudinal strain; HF: heart failure; LV: left ventricle/cular; LVSD: left ventricular systolic dysfunction; MACE: major adverse cardiovascular events; MI: myocardial infarction; RA: rheumatoid arthritis.

**Table S3: Univariate and adjusted (model B) Cox regression analyses for primary and secondary outcomes related to subclinical LVSD.**

| **Subclinical LVSD**  **(GLS > -18%)** | **Univariate Cox regression** | | | **Adjusted Cox regression (Model B^#^)** | | |
| --- | --- | --- | --- | --- | --- | --- |
|  | **HR** | **95% CI** | ***p-value*** | **HR** | **95% CI** | ***p-value*** |
| **Primary outcome** |  |  |  |  |  |  |
| MACE | **5.52** | **(2.39-12.8)** | **<0.001** | **3.91** | **(1.59-9.60)** | **0.003** |
| **Secondary outcomes** |  |  |  |  |  |  |
| 3P-MACE | **6.85** | **(2.34-20.1)** | **<0.001** | **5.02** | **(1.59-15.8)** | **0.006** |
| HF hosp. / CV death | **6.09** | **(2.04-18.2)** | **0.001** | **3.65** | **(1.11-12.1)** | **0.034** |
| HF hosp. | **4.24** | **(1.14-15.8)** | **0.031** | 3.10 | (0.75-12.7) | 0.117 |
| MI | 1.73 | (0.16-19.1) | 0.656 | 1.86 | (0.15-22.5) | 0.627 |
| CV death | **16.1** | **(1.88-138.1)** | **0.011** | 7.69 | (0.75-79.0) | 0.086 |
| All-cause mortality | **3.55** | **(1.44-8.76)** | **0.006** | 1.51 | (0.52-4.35) | 0.445 |
| Distance in 6MWT (↓ 50 m)* | **1.22** | **(1.05-1.49)** | **0.011** | 1.11* | (0.90-1.35) | 0.307 |

Table S3 presents univariate and adjusted Cox regression analyses, including respective HR, for primary and secondary outcomes related to subclinical LVSD (GLS > -18%) in comparison to normal LV GLS (GLS ≤ -18%). The multivariate Cox regression (Model B) was adjusted for the following covariates^#^: age, sex, diabetes mellitus, RA duration, and eGFR.

MACE consists of a composite of MI, HF hospitalisation, stroke, or CV death.

3-Point MACE consists of a composite of MI, stroke, or CV death.

*Odds ratio per each less 50 m walked.

^#^Based on the model by Ferreira et al. [8] from the same cohort.

Significant associations (*p-value* < 0.05) are in bold.

Abbreviations: 6MWT: 6-min walk test (distance in meters); BMI: body mass index; CI: confidence interval; CV: cardiovascular; eGFR: estimated glomerular filtration rate; GLS: global longitudinal strain; HF: heart failure; HR: hazard ratio; hsTnT: high-sensitivity troponin T; LV: left ventricle/cular; LVSD: left ventricular systolic dysfunction; MACE: major adverse cardiovascular events; MI: myocardial infarction; RA: rheumatoid arthritis.

**Table S4: Univariate and multivariate Cox regression analyses** **for the occurrence of MACE in RA patients, categorised by LV GLS or diastolic function.**

| **MACE** | **Univariate Cox regression** | | | **Adjusted Cox regression** | | |
| --- | --- | --- | --- | --- | --- | --- |
|  | **HR** | **95% CI** | ***p-value*** | **HR** | **95% CI** | ***p-value*** |
| **LV GLS categories** |  |  |  |  |  |  |
| Normal GLS (≤ -18%) | Ref. | - | - | Ref. | - | - |
| Borderline GLS (-18% < GLS ≤ -16%) | **3.16** | **(1.06-9.44)** | **0.039** | 2.38 | (0.78-7.20) | 0.126 |
| Abnormal GLS (> -16%) | **9.47** | **(3.75-23.9)** | **<0.001** | **7.18** | **(2.55-20.3)** | **<0.001** |
| **Diastolic function** |  |  |  |  |  |  |
| Normal diastolic function | Ref. | - | - | Ref. | - | - |
| Indeterminate diastolic function | 2.70 | (0.96-7.57) | 0.059 | 1.95 | (0.68-5.59) | 0.215 |
| Diastolic dysfunction | 2.29 | (0.81-6.41) | 0.116 | 1.45 | (0.47-4.42) | 0.517 |

Table S4 presents univariate and adjusted Cox regression analyses, including respective HR, for the occurrence of MACE in RA patients, categorised by LV GLS or diastolic function. RA patients with abnormal LV GLS (> -16%) had an adjusted 7.2-fold higher risk of developing MACE (HR 7.18; 95% CI 2.55-20.3; *p*<0.001) compared to RA patients with normal LV GLS (≤ -18%). There were no significant differences between patients with normal LV GLS and those with borderline LV GLS (*p*=0.126). Regarding diastolic function, there were no significant differences in the occurrence of MACE between patients with normal diastolic function and those with indeterminate or diastolic dysfunction (*p*=0.215 and *p*=0.517, respectively).

MACE consists of a composite of MI, HF hospitalisation, stroke, or CV death.

Ref. indicates the reference category, which corresponds to normal LV GLS or normal diastolic function.

The adjustment model included the following covariates: age, hypertension, dyslipidaemia, BMI, and eGFR.

Significant associations (*p-value* < 0.05) are in bold.

Abbreviations: BMI: body mass index; CI: confidence interval; CV: cardiovascular; eGFR: estimated glomerular filtration rate; GLS: global longitudinal strain; HF: heart failure; HR: hazard ratio; LV: left ventricle/cular; MACE: major adverse cardiovascular events; MI: myocardial infarction; RA: rheumatoid arthritis.

**Supplementary Figure Legends**

**Figure S1: Flowchart of the study population.** CABG: coronary artery bypass graft; CAD: coronary artery disease; PCI: percutaneous coronary intervention.

**Figure S2: Distribution of RA patients across different LV GLS categories.** Fig. S2 depicts the distribution of the study population across different LV GLS categories. Among the 277 RA patients without known heart disease included in the analysis, 76% (*n*=210) exhibited normal LV GLS, 14% (*n*=39) had borderline LV GLS, and 10% (*n*=28) had abnormal LV GLS.

**Figure S3: Distribution of LV GLS categories in relation to diastolic function.** Fig. S3 illustrates that the prevalence of subclinical LVSD is higher among patients with diastolic dysfunction, although not reaching statistical significance (*p* = 0.073 – see Table 1).

**Figure S4: Kaplan-Meier survival curves, categorised by LV GLS, in relation to the occurrence of MACE in RA patients.** Kaplan-Meier survival curves depicting the impact of LV GLS on MACE in RA patients. Survival analysis showed significant differences (*p* < 0.001; log-rank test) in the occurrence of MACE between the different LV GLS categories during a 7-year period of follow-up.

**Figure S5: Cumulative MACE-free survival curves in RA patients, categorised by** **diastolic function.** Survival analysis showed no significant differences (*p* = 0.078; log-rank test) in the occurrence of MACE between the different diastolic function categories in RA patients during a 7-year period of follow-up.
